# State Medicine

**Published:** 1883-08

**Authors:** A. L. Gihon


### Selections.

---

ADDRESS BY DR. A. L. GIHON ON STATE MEDICINE. AMERICAN MEDICAL ASSOCIATION, Cleveland, June 6.

Superficial observers see in the question which has arisen in the State Medical Society, of New York, only an attempt to break down the guards which hedge in the kingship of our profession. They have, indeed, proclaimed afar that the time-honored traditions of the guild are to be ignored, and the right hand of fellowship given to the accursed unbeliever. A year has passed since this question arose, and the dignified body of which we form a part evaded meeting the issue face to face. A few voices were raised in explanation, but they were drowned in the cry: "Crucify him, crucify him! he breaks bread with a homœopath!" The august fathers of the Association frowned their displeasure, and the venerable puppets of antiquity were taken down from their dusty niches and displayed to exorcise this new demon of the nineteenth century. Outside the halls animated ex parte statements were circulated, and a thousand delegates went away believing that a few individuals in the city of New York, mainly specialists, for purposes of personal profit, were advocating the license to consult and confer with avowed homœopaths—this and nothing more. Since then the matter has acquired a newspaper notoriety, and it is blazoned to the world as a fact, that the delegates of the State and county medical societies of New York have disqualified themselves for association with us, because they have sanctioned the formal recognition of homœopaths in clinical conference—and the popular sympathy of the profession has been aroused against these mercenary innovators. It is, of course, the business of the Judicial

Council "to take cognizance of and decide all questions of an ethical or judicial character that may arise in connection with the Association;" but every member of the Association has an equal interest with these twenty-one in the inquiry into the causes which have induced some of the most exemplary members of the profession to their course. Is it, as alleged, that the code of ethics is an antiquated piece of verbosity? Does it really accomplish what it professed? Undoubtedly its purport was the exclusion from professional fellowship of all but those who are entitled to it by their intelligence, education, professional skill and acquirements, and that fearless probity that doth become a man. Has it done this? Does it do it to-day? Are there intelligent, educated, skillful, and upright men in the profession because of the code, or in spite of it? It is unquestionably true there are no homœopaths in the American Medical Association, but are there any allopaths there? Does it say "Brother" only to those who are fit to wear the mantle of the wise physician? These are questions for the Association to ask itself—and primarily it is for the State Medicine Section in its purview over medical education to discuss calmly, fearlessly, and thoroughly—and to go with its conclusions and announce them to the Association, however unpalatable or unpopular.

I do not propose at this time any formal criticism or arraignment of the code of ethics. Practically three lines only are the shibboleth which the elect are required to utter. "No one can be considered as a regular practitioner, or a fit associate in consultation, whose practice is based on an exclusive dogma." Prior to this it is stated that "a regular medical education is presumptive evidence of professional abilities and acquirements;" but you may read the fifteen pages of the code in vain for the definition of what constitutes "a regular medical education," and it is to this I now propose especially to limit my inquiry—whether, while straining at the gnat in the twelfth, thirteenth, and fourteenth lines of section 1, article 4, we have not swallowed a camel in the other eleven.

Before all, however, it is proper that I should satisfy you of my own right to thus sit in judgment, and for this I must first explain that the conditions for admission into the medical corps of the navy are not of any exceptional or exclusive character, beyond the limitation of age between a minimum of twenty-one and a maximum of twenty-six, but are merely such as should be fulfilled by any young man who is certified in classic diction, by seven or more distinguished professional elders, to be *doctus in arte medica*—learned in medicine. The pre-requisites of respectability of character and correctness of deportment established by testimonials from citizens of known probity ought not to be considered superfluous even for the matriculant, while the modest scholastic acquirements insisted upon, rarely extending beyond a rudimentary familiarity with ortography, grammar, geography, and arithmetic, are not a tittle of what ought to be necessary preliminaries to the study of the first bone. The possession of a degree is not required, as it undoubtedly ought to be, and would be, did it carry with it any assurance of professional qualification—the code of ethics to the contrary notwithstanding. Why a degree, however regular in origin, is not considered satisfactory evidence of professional qualification, I expect to abundantly illustrate in this paper. Furthermore, the catholic liberty to compete for any position under the government has not allowed the board to decline to accept the candidacy of any individual claiming the ability to practice our art, and accordingly we have examined graduates of eclectic colleges and homœopathic colleges, as well as those who did not graduate at any college, who were thus permitted to stand on the same footing with other possessors of sheets of parchment, containing their names in a form they would not recognize and in a language they could not interpret. Though all such *irregulars* have failed to pass on examination, it is but just to state that the files of the department will show that they had no lack of peers in ignorance among the alumni of institutions of the most renowned name and unassailable regularity ; and I fail to see how the board in any way countenanced these practitioners of any exclusive dogma, or lowered the prestige of our own exclusive possession of the only true faith, by consenting to demonstrate to these pretenders that they were really ignorant and incompetent.

I have selected from my notes samples of the replies of twenty-five candidates—*doctores*, all of them—men certified to be learned in that noblest of all the sciences which engage the human intellect. I have endeavored to range them over as wide a field of inquiry as possible to show that it was not on abstruse or theoretical points on which the deficiencies were manifested. I have taken them from the graduates of schools in every section of the country, and those of established reputation, in order to prove that no one school, however exalted its rank, can claim exemption from the charge of having graduated grossly illiterate and incompetent men; and that diplomas have been sold, not cheaply and openly, as by Paine and his fellows, but still for a price, that price nominally two full courses of lectures—meaning two full sets of tickets, a matriculation and a graduation fee. I cannot, of course, say how many of these candidates actually fulfilled their implied obligation to attend lectures by sitting wearily on benches listening to discourses that must have been totally unintelligible to most of them, because I am not unjust enough to intimate that their professors did not honestly and faithfully fulfill their part by lecturing, but I do declare that their words fell like idle wind upon hearers such as these, and that the dolt who sat there throughout the course, or a part of it, or who did not even sit there but bought the ticket, issued forth, a full fledged doctor, the peer of the brightest, most industrious and most zealous student in the class. Some of the revelations of these examinations would be amusing were it not for the lamentable fact that many of their authors have for years been intrusted with the lives of their fellow beings, and this by the authority of the most respectable regular medical colleges in the United States.

To begin with the original seat of medical learning in this country. A graduate of one of the great schools, whose diploma was five years old, who had been recently prepared for examination, and whose demeanor evidenced the high opinion he held of his own abilities, stated that "the corpus callosum is that part of the dura mater which separates the cerebrum from the cerebellum"; that "the vertebral artery communicates with the cavernous sinus"; that "the membranous part of the urethra is below the triangular ligament, which is an extension of Gimbernat's ligament"; and that "pneumothrax is a collection of pus in the chest."

A second from the same institution, four years graduated, informed us that "the principal muscles of the larynx are the vocal cords"; that "the one-seventieth of a drop of hydrocyanic acid is the dose for an adult, and the tenth of that the proper quantity for a child"; that "the boiling point of Fahrenheit is about 300 degrees, that of the Centigrade scale is not so high, and that of Reaumer higher"; that he "could not tell what the zero of Fahrenheit was was without having the other scales" before him; and that "the normal temperature of the human body is from 112° to 140°."

From a sister school in the same city came a gentleman well provided with testimonials of his social, literary and professional qualifications for the Naval Medical Corps, who stated that he did not remember the country nor the language of Hippocrates," that "Galen lived before him," and "Virgil died in the fifteenth century of the Christian era."

It was a graduate of this same school, who, indeed, brought letters to me from an esteemed friend, and deservedly distinguished physician in Philadelphia, who, having absented himself several days during his examination, on his return announced to the president that he had been ill with "cholera infantum," and gravely reiterated the statement, when, with some astonishment, I repeated the inquiry. * * *

A representative from a New England school announced that "phosphorus burns and makes nitrogen gas;" that "the crepitant rhoncus occurs whenever a membrane becomes dry;" and as a part of his academic education, that "Plymouth was settled by Columbus, who gave the town its name;" and that each State and Territory has one Senator, who is a man of great influence; one of this great influential body having, in fact, indorsed this learned doctor's competency to assume the care and responsibility of the sick of the Navy. * * * *

A graduate of another deservedly celebrated New York school announced that "Galen introduced vaccination or inoculation in the seventeenth century," and that Aristotle was some ancient philosopher who wrote a book," probably intending that "'master-piece ' " which naughty youths delight to read, while still another New England M.D. explained that "the conditions which would render bronchomoty necessary would be where a foreign body has lodged in one of the bronchial tubes. I know of but one method of performing it, and it is to cut down carefully, being guided by the physical signs and your anatomy, and after severing the bronchial tube in a longitudinal direction, I would remove the foreign body, and carefully close up the wound;" that an "astringent is a medicine which corrugates or contracts the tissues;" and that "to determine accurately whether a person be dead or not, a knowledge of anatomy and physiology is essential. One of the surest signs of death is complete cessation of the heart's action, accompanied with a cold surface of the skin, a blueness of the lips and fingers, and a *coldness of the teeth.*"

These examples were not especially collected for my present purpose. I have merely used such as I had casually jotted down on my memoranda. The notes of my colleagues, and the archives of the Burean of Medicine and Surgery can supply thousands of instances of professional incapacity where I have noted a hundred, and my successors of to-day upon the Board are having the same experience. Within a week, one wrote me that "the comic opera and minstrel performances are nothing compared to the Board entertainments." Ridiculous errors in orthography, inexcusable in a day laborer in this land of boasted educational facilities, are so common that one might believe correct spelling to be a refinement of culture. "What the hell has spelling to do with the practice of medicine?" demanded an irate member of Congress of the chief of the Bureau, Dr. Whelan, on being shown his protege's written work, and doubtless so thought the trustees of a charitable institution, widely known, who admitted into its resident medical staff, after competitive examination, a gentleman, the graduate from a university, who, two years subsequently, gave the board the following written evidence of his literary acquirements: *immergencies*, *attact*, *thare*, *redy*, *vigitable*, *xertion*, *urin*, *pluracy*, *deadly knight-shade*, and *expultion of its contence.* Without questioning that there are intellectual giants, who achieve greatness in certain careers, though unable to spell at all, it may be doubted whether the average mind accustomed to write *scas-*

*sity*, and *orful*, and *inturlec*, and *aurora epeleptica*, in total ignoranbe of their etymological significance, can ever succeed in comprehending the language which has come to be technical in every department of our science, and only errors such as these, and not mere phonetic mistakes, have, of course, been taken into account. Indeed, we did not decline to examine the candidate who addressed his letter to the *Navel mediclebord*, and subscribed himself, *Yours respectively.*

Of 1,141 candidates for the medical corps in the thirty years since 1853, only three hundred and seventy, or about thirty-two per cent., have been accepted. * * * *

Mr. Chairman: The time has come when this Association must be up and doing. A few medical schools have undertaken the reform, but the movement that has been inaugurated, notably by Harvard, will avail little, so long as the Association unconcernedly witnesses and indirectly countenances the wholesale manufacture of doctors elsewhere—by accepting their membership without question of their competence. The time has come when something more than paper bulwarks shall be considered defense for our orthodox stronghold, and proper partitions sufficient to separate the sheep from the goats. The time has come for us to act, and it is eminently proper that the section in State medicine shall be the scene of action. The discussion in New York has made it impossible for us to remain indifferent spectators. I trust that no member of this section has been misled into believing that any of the distinguished men who have taken part in this agitation have asked for anything that involves, in the least degree, any concession to the claims of homeœpathy, allopathy, or any other exclusive dogma; that none of them have suggested, advocated, or desired any arrangement that can provide for or permit the joint treatment in any case by themselves and homeœpaths, or any other paths. They have, however, claimed the right to give their opinion to any one who asks for it and is willing to pay for it—to tell any sick person what he thinks of his case, and what he considers ought to be done. In this, they give no sanction to the "irregular," whoever he may be, who has sought their advice, even though this irregular may be even wiser than that regular who castrates in orchitis, and gives his puerperal woman a Russian bath. The very fact of his advice having been sought is an admission on the part of the irregular that he has done wrong or knows not what to do, and if, as alleged, these irregulars have not sought these consultations, then none will be, and the storm is but a tempest in a teapot. It is for you, however, to determine decisively whether you will place under the ban the man whose only offense has been to say to one of these irregulars, "You are doing wrong," or will exclude from the ranks of the profession that one of your distinguished colleagues in New York, who, when called in consultation in a case of difficult labor, requiring instrumental interference, did not stop to examine the diploma of the attending physician, but went to work, finding him an exceedingly expert assistant, and learned, when the patient had been rescued from danger, that he had been co-operating with a homeœpath; or will threaten the young road surgeon, who, at the close of the meeting at St. Paul, asked me whether he should operate in a case of caries of the tibia in a poor woman at a station where the only physician was a homeœpath, "Do it if you dare. You shall never cross our threshold if you do. Let her die first."

But I am only repeating a trite, thrice told tale. The necessity for reform in medical education is undeniable. Let us begin the work of reformation. If the American Medical Association is something more than a convivial assembly—if it is sitting here as a senate in serious delibration as to the interests of the profession—it can no longer ignore this question. Years ago your excellent committee, Drs. Davis, Toner, and Weatherby, and the committee of the Medical Society of the State of New York, Drs. Squibb, Eliot, and Bradford, provided for raising the stand- of preliminary education required of medical students by compelling preceptors to exclude from their offices all who fail to receive the certificate of a properly constituted board of censors, but the colleges must be coerced into doing their share. Harvard and the University of Pennsylvania will fight a hopeless battle if they are without allies.

It is for you to say to every one of these great schools and famous teachers, as well as to the petty schools andtheir tyros in teaching, you must, and at once, set the example of reform; require a preliminary, scholastic training of your matriculants; appoint examining boards who shall have no pecuniary interest in the size of your graduating classes; make your examinations in part written, to remain on record as evidence of the candidate's ability or lack of it; and insist upon a practical familiarity of the novice with the tools of his art, its drugs, and apparatus and instruments. For a while there will be a falling off in revenue, but the code of ethics denounces the prostitution of our calling for sordid purposes. And what more mercenary spectacle can there be than that witnessed at the close of a college session, when, with blessings and music, and big boquets, a new battallion of doctors marches forth commissioned to guard the most solemn responsibilities humanity offers.

In furtherence of these objects, I beg to offer the following resolutions, to be referred to the Association, should the section on State medicine give them its approval.

*Resolved*, That the section in State medicine urges upon the Association the necessity for at once taking steps to exclude unqualified members from the profession by refusing fellowship to illiterate, ignorant and incompetent graduates.

*Resolved*, That the Association be recommended to authorized the section in State medicine to act as a standing committee on medical education, the several elected members being required to communicate without delay (I.) with the several State medical societies and the legislatures of the States they respectively represent, with the object of creating State boards of medical examiners, where such are not already in existence, whose certificate shall be necessary to the issue of a license to practice medicine in that State; and (II.) with the authorities of every regularly organized medical college in the State which has not already taken such action, urging upon them, first, the requirement of a proper preliminary education of matriculants, to embrace, at least, a knowledge of English othography and grammar, the etymology of the more common Greek and Latin derivations, and the fundamental rules of arithmetic, to be ascertained by a written examination preserved for reference; and, second, greater care in ascertaining the fitness of candidates for a degree by making their final examination in part a written one, to be kept on record and accessible for inspection by the State board of medical examiners, board of censors of medical societies, or authorized persons requiring information as to the professional qualifications of graduates.

*Resolved*, That in the opinion of the American Medical Association, medical colleges should confer upon graduates the degree of *Batchelor in Medicine*, such graduates to be eligable to the degree of *Doctor of Medicine* at the end of three years, after having given satisfactory evidence of their qualification to the board of censors of the State medical society.

*Resolved*, That article 2 of the plan of organization of the American Medical Association be amended by this additional provisio:

*Provided*, That every permanently organized State, county or district medical society entitled to representation in this Association shall be required to appoint a board of censors, who shall rigidly scrutinize the literary and professional qualifications of every candidate for membership therein, and hereafter no delegate shall be admitted to a seat in this Association who shall not have received the certificate of such a board of censors, or of a State or National board of medical examiners.

Drs. Rauch, of Illinois; Billings of the United States Army; Tuckerman, of Ohio; Hibbard, of Indiana; Hicks, of Pennsylvania; Sheean, of New York; and Bush, of Delaware, discussed the resolutions, when further action was postponed until to-morrow.

